# Effects of Incentives on Adherence to a Web-Based Intervention Promoting Physical Activity: Naturalistic Study

**DOI:** 10.2196/18338

**Published:** 2020-07-30

**Authors:** Ramona Wurst, Anja Maliezefski, Christina Ramsenthaler, Judith Brame, Reinhard Fuchs

**Affiliations:** 1 Department of Sport and Sport Science University of Freiburg Freiburg Germany; 2 IGEL-M GbR Bad Oldesloe Germany

**Keywords:** internet-based intervention, adherence, incentive, reward, mHealth, eHealth, exercise, dropout rate, usage, attrition, telemedicine

## Abstract

**Background:**

Despite many advantages of web-based health behavior interventions such as wide accessibility or low costs, these interventions are often accompanied by high attrition rates, particularly in usage under real-life conditions. It would therefore be helpful to implement strategies such as the use of financial incentives to motivate program participation and increase adherence.

**Objective:**

This naturalistic study examined real-life usage data of a 12-week web-based physical activity (PA) intervention (Fitness Coach) among insurants who participated in an additional incentive program (incentive group) and those who did not (nonincentive group). Users in the incentive group had the perspective of receiving €30 (about US $33) cash back at the end of the intervention.

**Methods:**

Registration and real-life usage data as part of routine data management and evaluation of the Fitness Coach were analyzed between September 2016 and June 2018. Depending on the duration of use and the weekly recording of tasks, 4 adherence groups (low, occasional, strong, and complete adherence) were defined. Demographic characteristics were collected by a self-reported questionnaire at registration. We analyzed baseline predictors and moderators of complete adherence such as participation in the program, age, gender, and BMI using binary logistic regressions.

**Results:**

A total of 18,613 eligible persons registered for the intervention. Of these, 15,482 users chose to participate in the incentive program (incentive group): mean age 42.4 (SD 14.4) years, mean BMI 24.5 (SD 4.0) kg/m^2^, median (IQR) BMI 23.8 (21.7-26.4) kg/m^2^; 65.12% (10,082/15,482) female; and 3131 users decided not to use the incentive program (nonincentive group): mean age 40.7 (SD 13.4) years, mean BMI 26.2 (SD 5.0) kg/m^2^, median BMI 25.3 (IQR 22.6-28.7) kg/m^2^; 72.18% (2260/3131) female. At the end of the intervention, participants in the incentive program group showed 4.8 times higher complete adherence rates than those in the nonincentive program group (39.2% vs 8.1%), also yielding significantly higher odds to complete the intervention (odds ratio [OR] 12.638) for the incentive program group. Gender significantly moderated the effect with men in the incentive group showing higher odds to be completely adherent than women overall and men in the nonincentive group (OR 1.761). Furthermore, older age and male gender were significant predictors of complete adherence for all participants, whereas BMI did not predict intervention completion.

**Conclusions:**

This is the first naturalistic study in the field of web-based PA interventions that shows the potential of even small financial incentives to increase program adherence. Male users, in particular, seem to be strongly motivated by incentives to complete the intervention. Based on these findings, health care providers can use differentiated incentive systems to increase regular participation in web-based PA interventions.

## Introduction

Promoting a healthy lifestyle, especially reducing sedentary behavior and increasing physical activity (PA), is crucial in the prevention of chronic diseases such as cardiovascular disease, type 2 diabetes, breast and colon cancer as well as hypertension [1]. Persons who follow the PA recommendations of the World Health Organization [2] show a 27% decreased risk of mortality [3]. Besides numerous face-to-face PA interventions [4,5], electronic health (eHealth) and mobile health (mHealth) interventions have become increasingly popular to promote health behavior change such as increasing PA levels [6]. These interventions have the potential to reach large numbers of people in a cost- and time-efficient way [6]. Testing the effectiveness of internet interventions for behavior change, in particular for promoting PA, has yielded positive results despite small effect sizes with standardized mean differences ranging from 0.14 to 0.20 for self-reported PA [7-10].

Based on this evidence, many stakeholders in health care including health insurers have developed web-based interventions promoting PA as a public health approach [11,12]. However, *program adherence* to web-based interventions, that is, using the program as intended by the developers over the course of the intervention, has always been a major challenge (law of attrition [13]). Despite the positive effects on PA described above, internet interventions suffer from high dropout rates of up to 80% at the end of the intervention [14-16]. This lack of program adherence is likely to negatively influence *behavior adherence* [17,18], that is, the maintenance of newly acquired PA behavior in the real life after the program is finished.

To increase program and behavior adherence of web-based interventions, many providers such as health insurers revert to financial incentives such as cash, bonus points, or charity rewards [12,19]. The rationale behind using such external rewards to change health behavior is based on the assumption that this strategy may trigger an increasingly intrinsic motivation for PA that stabilizes the behavior even after the incentive removal [19]. In *face-to-face* settings, financial incentives have been shown to increase real-life exercise adherence (behavior adherence) in the short term and partially even in the long term among children, adolescents, and adults [20,21]. Similar results have also been found for *web-based* interventions with increased PA levels reported by incentivized groups after the intervention [12,19,22-24]. It appears that those incentives do not need to be of high value. Small financial incentives of about US $1.50 per day or collecting loyalty points for coupons have been shown to substantially impact on uptake rates of eHealth or mHealth interventions [12,25,26].

Only a few studies using incentives for *web-based* PA interventions report *program adherence* rates for incentivized groups [12,23]. Results are equivocal: A web-based step count intervention for patients with ischemic heart disease lasting 24 weeks found a substantial difference between the incentivized group and the control group regarding program adherence (62.1% vs 51.2%) [23]. The incentive group was given US $14 per week and they could lose US $2 per day if they did not achieve their step goals. The results of this loss-framed incentive study point toward a beneficial effect of financial incentives to increase program adherence in web-based PA interventions. In another randomized controlled trial with insurants in Switzerland, differences between the incentivized and control groups were not significant after 3 months. However, program adherence rates were rather high in both groups with 92.1% in the incentive group (US $10 per month) and 90.5% in the control group [12]. Both groups were given discount to an activity tracker and they had to wear the tracker every day which probably also motivated the control group to participate regularly.

Data on program adherence rates of *app-based* PA interventions (daily step counts) are provided by the Carrot Rewards App Study (N=32,229) in which all users of the app collected loyalty points that could be redeemed for travel, groceries, or movies [22]. Results revealed that 61.9% of those who had initially registered as users completed the intervention after 12 weeks [22]. Unfortunately, there was no control group without incentives in the study, and therefore, their specific effect remains unclear here. From other app-based PA interventions, however, it is known that program adherence is markedly lower when no incentives are provided. For instance, in a naturalistic study by Guertler and colleagues [15], only 25% continued to use the PA promotion app after 6 weeks.

In sum, using incentives in web- or app-based PA interventions has the potential to increase program adherence in clinical trials. However, little is known about the effects of incentives on program adherence in real-life settings. All of the above reported results on program adherence were taken from studies in which users knew that they were part of a study and their data were tracked for study purposes. There is evidence that usage behavior differs substantially between people who use the programs on their own (ie, open-access users) and those who know that they are participants of clinical trials [6,27,28]. For example, Wanner and colleagues [27] showed that only 25.8% of the open-access users visited the PA internet intervention repeatedly compared with 67.3% of the trial participants [27]. This result reflects the high discrepancy of trial and real-life usage and points toward the need for naturalistic studies investigating the effects of incentives among open-access participants [6].

Besides real-life adherence rates, there is also no research on the factors that moderate program adherence in web- or app-based PA interventions using financial incentives. From studies without incentives, we know that age and gender may be significant predictors of PA program adherence. Thus, older age groups have been shown to participate more frequently than younger adults in web-based PA interventions with odds ratios (ORs) ranging between 1.02 and 2.61 [15,27,29,30]. Regarding gender, results are more inconsistent. Whereas some reviews and studies revealed that women have a higher chance to complete an internet-based behavior change intervention (OR 2.24) [14,29], other investigations demonstrated that men use such interventions more continuously (OR 1.2; reduced hazard ratio to drop out 0.85) [15,27]. Studies that also included BMI in their analyses found that this parameter did not predict program adherence [27,29,31,32]. Given these age and gender differences in web-based program adherence, it is interesting to see how these differences are affected by the application of incentives.

This study’s purpose was to expand upon previous research about incentives in web-based PA interventions by examining naturalistic data (ie, real-life usage data) of the largest health insurance company in Germany. Members interested in the web-based intervention *Fitness Coach* could choose to participate in an additional incentive program at the beginning of the intervention. Based on earlier studies [22], we hypothesized that real-life adherence would be higher in the incentive than in the nonincentive group. Furthermore, we considered the effects of moderators and predictors such as age, gender, and BMI on program adherence. This is the first study to examine the role of incentives for program adherence to a web-based PA intervention in a naturalistic, large-scale study of over 18,000 participants.

## Methods

### Study Design, Setting, and Participants

This investigation is a naturalistic study examining the real-life usage data of open-access users who participated in a web-based intervention called *Fitness Coach*. Naturalistic (observational) studies do not “involve any intervention [...] on the part of the investigator” [33] and the data are not affected by the actions of researchers [34]. Using such a naturalistic study design might help us better understand real-life program adherence to web-based PA interventions. We followed the Strengthening the Reporting of Observational Studies in Epidemiology (STROBE) guidelines [35].

This study includes usage data from September 2016 to June 2018 of the Fitness Coach intervention provided by a large German health insurance company. The Fitness Coach intervention was freely accessible to everyone in Germany, but only insured persons could participate in the incentive program and noninsured persons could just use the intervention for at least four weeks. Most of the users in this study were members of the insurance company providing the intervention. In the first session of the Fitness Coach, they were screened for their suitability to exercise with the Physical Activity Readiness Questionnaire (PAR-Q +) [36]. Exclusion criteria were being younger than 18 years or being pregnant. If users met one or both exclusion criteria, they were advised to ask a physician about participating in the program.

All data entered by the open-access users including information about website use were collected and stored under a pseudonym by Vilua Healthcare GmbH (Berlin, Germany). Prior to the intervention, all users had to sign a data protection declaration in which they were informed that all data obtained could be further analyzed for research purposes. The Guidelines of the Declaration of Helsinki were followed. Given the retrospective examination of the real-life usage data, no ethical approval was obtained prior to data collection.

### Intervention

The Fitness Coach is a 12-week long, tailored, and technically guided internet intervention promoting PA. It is based on the MoVo concept consisting of motivational and volitional strategies [37] as well as training progression concepts [38]. The intervention was developed in 2005 by an insurance company in Germany and was relaunched in September 2016 in an updated version [11]. All users of the intervention receive an individualized training program tailored to their personal goals and daily life. This consists of training videos that match the person’s goals, that is, becoming stronger, improving one’s endurance, or becoming more flexible (Multimedia Appendices 1 and 2). Users also receive psychological input to help them initiate and maintain their behavior change. The intervention contains multiple behavior change techniques such as goal setting, action planning, self-monitoring, and feedback as well as coping planning [39].

### Incentive Program

As a form of primary prevention, health insurance companies in Germany are legally obliged to use incentives for promoting healthy behavior (eg, increasing PA and reducing weight). There is also evidence that investing approximately €30 (about US $33) per person and per year in incentives for health behavior change reduces the overall costs for the insurance company by approximately €100 (about US $112) per person and per year [40,41]. Based on these findings, all users of the web-based intervention who were insured by the insurance company could register for the incentive program at the beginning of the intervention. In order to receive the incentive, the users had to log the completion of at least three tasks (eg, strength, endurance, or mobility exercises; evaluating one’s well-being; reading background information) per week in 10 of 12 weeks. In case of adherence to the intervention, they received 500 credit points on their health insurance account. Upon the collection of 500 further credit points within 1 year (earned via membership in sports clubs or gyms, regular medical check-ups, or participation at a sporting event), they could choose between €30 (about US $33) cash back and €60 (about US $68) health credit. This health credit could be used for additional medical and health services such as professional tooth cleaning, acupuncture, osteopathy, and fees for gyms or sports clubs.

### Variables and Statistical Analyses

**Figure 1:**
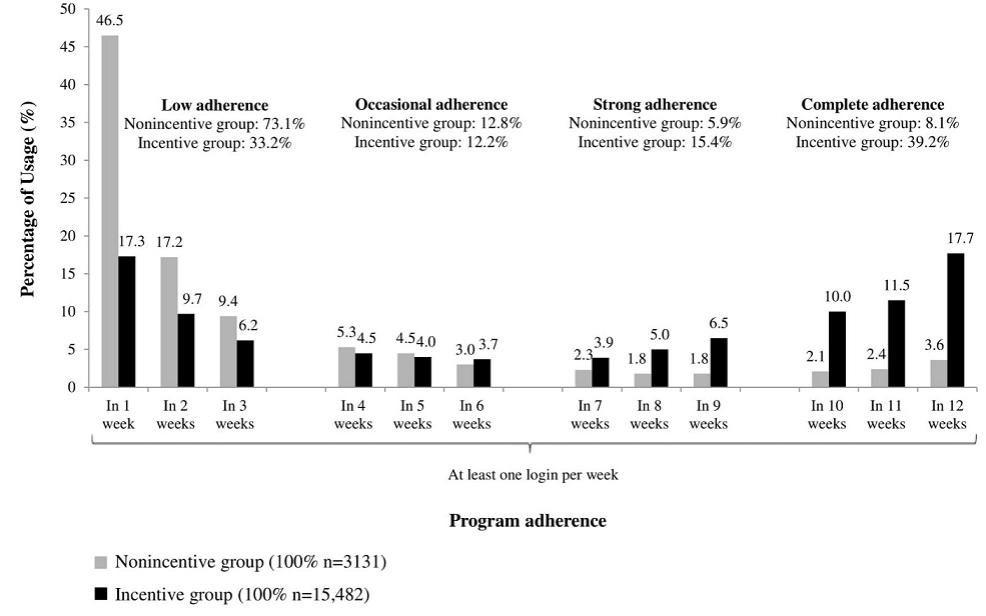
Percentage of Fitness Coach usage over 12 weeks divided into 4 adherence groups (N = 18,613).

Demographic characteristics of all users including age, gender, and BMI were obtained from the self-report questionnaire included in the first session of the intervention. Each login to the intervention per day was captured, reported as mean (SD), resulting in a minimum number of 1 and a maximum number of 84 logins over the 12-week intervention span (16.9 [SD 16.8]). In order to categorize adherence, login data were summarized per week. If a user had at least one login per week with at least three logged tasks in the week, the user was adherent and participation in that week was defined as successful [42]. Based on the health insurance company’s definition of successful participation (ie, ≥10 weeks), we classified the number of weeks with successful participation into 4 categories or adherence groups:

The *low adherence* group included all users participating for 1-3 weeks during the 12-week intervention span. *Occasional adherence* was defined as participation between 4 and 6 weeks. If users had 7-9 weeks of successful participation, we categorized them as *strongly adherent*. All persons completing 10 or more weeks successfully were defined as the *complete adherence* group (Figure 1). The last group was incentivized by the health insurance company.

Given the naturalistic sample, group sizes differ substantially in all 4 adherence groups as well as in the incentive and nonincentive groups (Table 1). We compared demographic variables between incentive and nonincentive groups using two-tailed Welch *t* tests and chi-square tests.

To investigate which characteristics would increase the chance of complete adherence and thus allowing the users to receive the incentive, binary logistic regressions were conducted using SPSS Statistics version 25 (IBM Corporation). We combined the first 3 adherence groups (low, occasional, and strong; =0) and compared them with the complete adherence group (=1) to best display the incentive effect on adherence. Incentive participation (0=no incentive, 1=incentive), gender (0=female, 1=male), and age and BMI (both continuous variables) were considered as baseline predictors. Furthermore, the interactions of incentive × age, incentive × gender, and incentive × BMI were considered as moderators for the association between incentives and program adherence. There were no missing data for adherence, incentive participation, age, and gender. Because it was not mandatory to enter height and weight, BMI data were missing in 530 cases, resulting in a total number of 18,083 users included in the logistic regression. After checking for multicollinearity between incentive participation, age, gender, and BMI (variable inflation factors between 1.024 and 1.066), all demographic variables and interactions were included in the regression model. Model fit was judged using Nagelkerke *R*^2^. The level of significance was set at *P*<.05.

**Table 1:** Demographic characteristics of the sample, grouped by adherence and incentive status.

Groups and characteristics					Nonincentive		Incentive
**Overall**							
	N				3131		15,482
	Age (years), mean (SD)				40.7 (13.4)		42.4 (14.4)
	BMI (kg/m^2^), mean (SD)				26.2 (5.0)		24.5 (4.0)
	BMI (kg/m^2^), median (IQR)				25.3 (22.6-28.7)		23.8 (21.7-26.4)
	**Gender**						
			Female, n (%)	2260 (72.18)		10,082 (65.12)	
			Male, n (%)	871 (27.82)		5400 (34.88)	
**Low adherence**							
	n (%)				2290 (73.14)		5133 (33.15)
	Age (years), mean (SD)				39.9 (13.3)		40.2 (13.7)
	BMI (kg/m^2^), mean (SD)				26.2 (5.1)		24.9 (4.3)
	BMI (kg/m^2^), median (IQR)				25.1 (22.5-28.8)		24.0 (21.8-26.9)
	**Gender**						
			Female, n (%)	1639 (71.57)		3452 (67.25)	
			Male, n (%)	651 (28.43)		1681 (32.75)	
**Occasional adherence**							
	n (%)				401 (12.81)		1895 (12.24)
	Age (years), mean (SD)				42.9 (12.9)		41.4 (13.8)
	BMI (kg/m^2^), mean (SD)				26.4 (4.8)		24.6 (4.1)
	BMI (kg/m^2^), median (IQR)				26.0 (22.7-29.0)		24.0 (21.7-26.7)
	**Gender**						
			Female, n (%)	288 (71.82)		1281 (67.60)	
			Male, n (%)	113 (28.17)		614 (32.40)	
**Strong adherence**							
	n (%)				187 (5.97)		2377 (15.35)
	Age (years), mean (SD)				42.0 (13.0)		41.9 (14.0)
	BMI (kg/m^2^), mean (SD)				25.9 (4.8)		24.4 (3.8)
	BMI (kg/m^2^), median (IQR)				25.1 (22.4-28.3)		23.8 (21.7-26.2)
	**Gender**						
			Female, n (%)	139 (74.33)		1542 (64.87)	
			Male, n (%)	48 (25.66)		835 (35.13)	
**Complete adherence**							
	n (%)				253 (8.08)		6077 (39.25)
	Age (years), mean (SD)				43.9 (14.2)		44.9 (14.3)
	BMI (kg/m^2^), mean (SD)				26.0 (4.9)		24.3 (3.8)
	BMI (kg/m^2^), median (IQR)				25.2 (22.6-28.4)		23.7 (21.6-26.0)
	**Gender**						
		Female, n (%)			194 (76.67)		3807 (62.65)
		Male, n (%)			59 (23.32)		2270 (37.35)

## Results

### Participants

**Figure 2:**
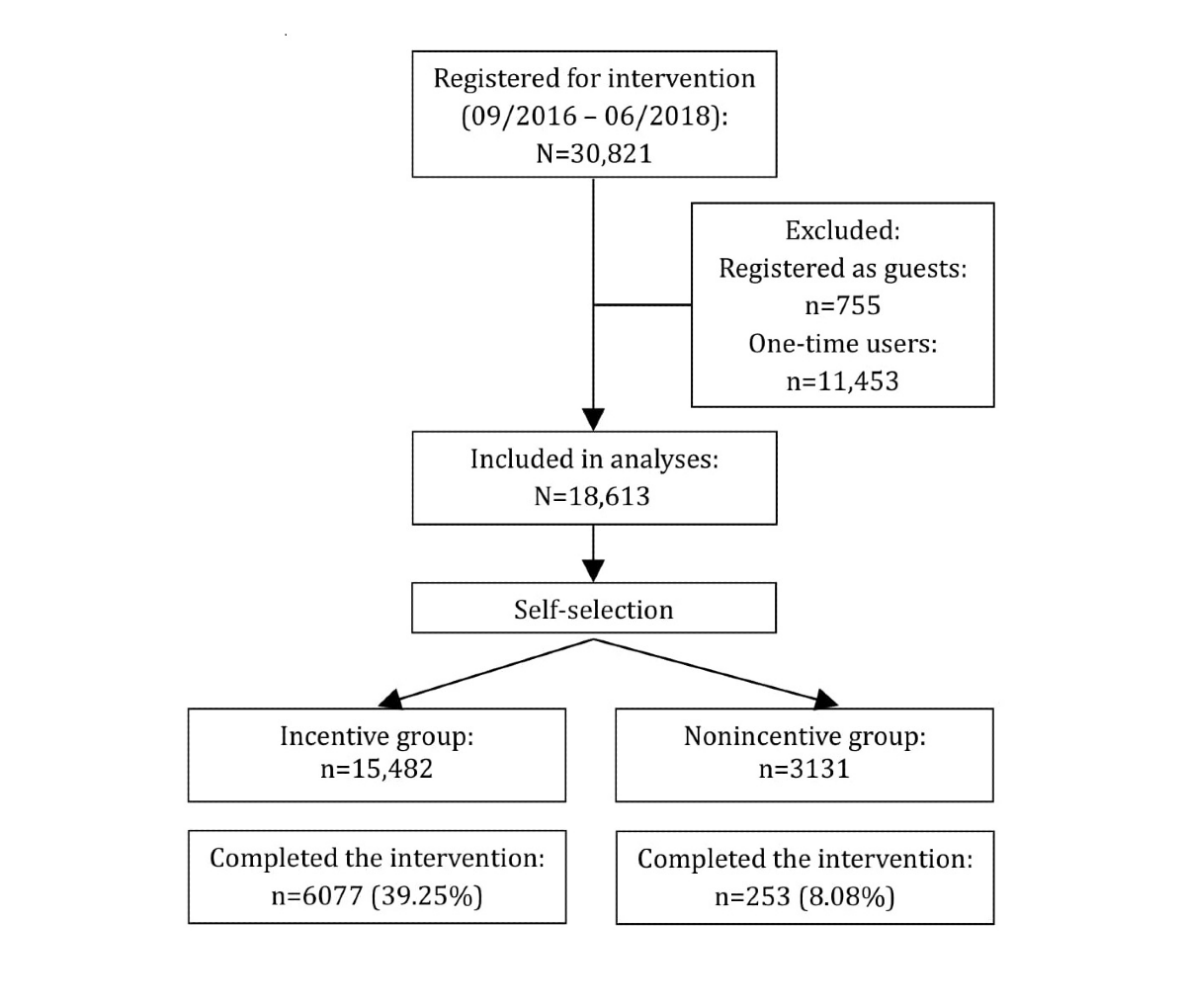
STROBE flow chart of Fitness Coach participants.

From September 2016 to June 2018, a total of 30,821 persons registered for the Fitness Coach intervention. Of these, 755 (2.45%) registered as guests who were not insured by the health insurance company and were therefore unable to participate in the incentive program. In addition, there were 11,453 (37.16%) insured persons who visited the internet intervention only once and had no further login. All guests and one-time users were excluded from further analyses, so the demographic analyses included the total number of 18,613 insured users (Figure 2).

Demographic characteristics differed significantly between the incentive and nonincentive groups. Age (*P*<.001), BMI (*P*<.001), and gender (*P*<.001) differences indicated that older and more male users with a lower BMI register for the incentive (Table 1). Table 1 also displays the total sample size for each group at the start and at the end of the intervention (complete adherence).

### Participation and Attrition Rates

In total, 34.01% of users (6330/18,613) were completely adherent (Figure 2). Among those complete adherers, the participation rate in the incentive group was 4.8 times higher than that in the nonincentive group: 39.25% (6077/15,482) of all members in the incentive group versus 8.08% (253/3131) of all members in the nonincentive group. By contrast, 73.14% (2290/3131) of members in the nonincentive group were classified as being lowly adherent, which means participating in less than 4 out of 12 weeks of the intervention, in comparison to 33.15% (5133/15,482) of members in the incentive group. The overall nonusage attrition rate (low, occasional, and strong adherence combined) in the nonincentive group reached 91.92% (2878/3131) compared with 60.75% (9405/15,482) in the incentive group (Table 1). The cutoff point of nonusage attrition was set at 9 or less successful intervention weeks.

### Predictors of Intervention Completion

Binary logistic regressions revealed that participating in the incentive program was a statistically significant predictor (*P*<.001) at baseline for completing the intervention (OR 12.638, 95% CI 5.614-28.449; Table 2). There were also moderating effects on the association between incentive participation and program adherence. Male users in the incentive group showed significantly higher odds to complete the intervention than female users overall and male users in the nonincentive group (OR 1.761, 95% CI 1.272-2.438). By contrast, the interactions between the incentive group and age or BMI did not yield significant odds for intervention completion.

Without taking the financial incentive participation into account (main effect), both age (*P*<.001) and gender (*P*=.017) proved to be significant baseline predictors of complete adherence to the Fitness Coach intervention for all users (Table 2), with older users having higher odds of Fitness Coach completion than younger ones (OR 1.023, 95% CI 1.013-1.033) and women having lower odds to be completely adherent than men (OR 0.679, 95% CI 0.494-0.933).

**Table 2:** Predictors of complete adherence (N=18,083).^a^

Characteristics	Logit odds	Standard error	Wald χ^2^	*P* value	Odds ratio	95% CI for odds ratio	
						Lower	Upper
Intercept	–2.847	0.398	51.046	<.001	0.058		
Incentive program	2.537	0.414	37.545	<.001	12.638	5.614	28.449
Gender	–0.387	0.162	5.712	.017	0.679	0.494	0.933
Age	0.022	0.005	19.594	<.001	1.023	1.013	1.033
BMI	–0.017	0.014	1.503	.220	0.983	0.956	1.010
Gender × Incentive	0.566	0.166	11.608	.001	1.761	1.272	2.438
BMI × Incentive	–0.027	0.015	3.205	.073	0.974	0.946	1.003
Age × Incentive	–0.001	0.005	0.072	.788	0.999	0.988	1.009

^a^Reference category is low, occasional, and strong adherence groups combined; Nagelkerke *R*^2^ (0.130), *df* (degrees of freedom) for all=1.

## Discussion

### Principal Findings

This large-scale, naturalistic study with a nationwide sample of 18,613 users examined the effect of an incentive program and its moderators on program adherence to a web-based PA intervention. Participants were members of the largest German health insurance who either decided to take part in an additional incentive program at the beginning of the intervention or rejected the use of the incentive program. Results showed that complete program adherence was 4.8 times higher in the incentive group than in the nonincentive group. In line with this finding, binary logistic regressions revealed incentive program participation to be a strong and significant predictor for complete adherence (OR 12.638, 95% CI 5.614-28.449, *P*<.001). This incentive effect on program adherence was moderated by gender, but not by BMI and age. Males in the incentive group showed the highest odds for program completion. Furthermore, gender and age also exhibited significant main effects on program adherence: being male and being of older age were associated with higher program adherence. However, BMI was not a significant predictor of program adherence.

Our findings demonstrate that even a small incentive (chance of €30 [about US $33] cash back at the end of the intervention) was able to enhance program adherence by almost five times, emphasizing the important motivating role of external rewards, at least in the initial phase of a new health behavior [19,39,43]. This result gains in importance because it is based on real-life usage data, which allows for a higher ecological validity. To the best of our knowledge, this is the first study investigating the effects of incentives on program adherence in a web-based PA intervention on the basis of naturalistic data. Therefore, no direct parallels can be drawn with data from the literature. However, comparisons can be made with similar real-life studies in which no incentives were used [27,28]. Those studies reported program adherence rates of 10%-25.8%. Compared with these findings, the program adherence rate of our nonincentive group (8.08%, 253/3131) is at the lower end of this range. By contrast, the program adherence rate of our incentive group (39.25%, 6077/15,482) substantially exceeded the program adherence rates of those two studies, suggesting once again that incentives might make a clear difference in the compliance with web-based PA interventions.

This study also found a *moderating effect* of gender on the association between incentive participation and program adherence. Men in the incentive group had significantly higher odds to complete the intervention than female users overall and male users in the nonincentive group, indicating that financial incentives may appeal particularly to men. This is supported by Czap and colleagues [44] who reported men to be more attracted to financial incentives than to other nonfinancial nudges. Given this clear gender difference in the motivational power of financial incentives, future research could investigate more closely which equivalent incentives might be more appealing to women.

By looking at the *main effects* of age on program adherence, we found older users to be significantly more inclined to complete the intervention than their younger counterparts, yet with a small OR of 1.023 (95% CI 1.013-1.033; *P*<.001). This result is well known from the literature, which indicates that older users have higher chances to be adherent than younger users [15,27,29,30]. It appears that on average users in their mid-40s are the most adherent ones to the web-based PA intervention. Regarding the main effect of gender on program adherence, there were more women than men participating in the Fitness Coach, suggesting that improving one’s fitness via internet appeals at first sight to women. This is in line with previous research reporting higher uptake rates of web-based PA interventions among women [6,8]. However, if we do not look at uptake rates but at adherence rates, the picture is quite different: women in our sample showed significantly lower odds to be completely adherent than men. There are several studies which support the finding that men are more adherent to these kinds of interventions [15,27]. For example, a large-scale, open-access evaluation from Switzerland yielded a significantly higher chance (OR 1.23) for men to participate repeatedly in a web-based intervention than for women [27].

The scope of this study was on *program adherence*; therefore, no definite conclusions can be drawn on the actual adherence to regular PA after the end of the 12-week intervention (behavior adherence). However, it is in the logic of time-limited behavior change programs that after finishing the program the newly acquired health behavior has to be continued in everyday life on one’s initiative. We cannot be certain how successful the health behavior change has been in the present sample, yet there is evidence that a higher level of program adherence also leads to sustained health behavior change in everyday life [17].

### Strengths and Limitations

This study is the first large-scale examination that investigated whether financial incentives increase program adherence to a web-based PA intervention under real-life usage conditions. Because of the naturalistic study design, the results of this investigation have high ecological validity.

However, some limitations need to be addressed. Internal validity might be threatened as the assessment of program adherence depends on self-reported participation behavior. There is also a risk that self-reported data on age and BMI may be distorted by socially desirable response tendencies or even worse, false data, to reveal as little information about oneself as possible. The study uses a naturalistic design with self-selection of participants into the incentive or nonincentive conditions. Given this design, it was not possible to randomize users into these two groups. Overall, both groups differed substantially regarding age, gender, and BMI with older users, more men, and users with lower BMI choosing to participate in the incentive program. It might be hypothesized that participants in the incentive group were more motivated from the beginning to use the program than users who did not register for the incentive.

Given the unequal sample sizes for incentive and nonincentive groups (15,482 vs 3131) as well as for the low, occasional, and strong adherence groups combined and the complete adherence group (12,283 vs 6330), the results of binary logistic regressions might be overestimated. This is reflected, for example, in very large CIs and statistical significance despite small OR (eg, OR_Age_ 1.023, 95% CI 1.013-1.033; *P*<.001). However, the decision for the large comparison group was made from a content perspective focusing on representativeness and external validity. The unbalanced analyses took account of the naturalistic data and better displayed how complete adherers differ from the other users who did not successfully finish the intervention. The logistic regression was also based on large sample size. While the unbalanced distribution might have affected the width of the confidence intervals, it has not affected the ORs in the logistic regression because there is enough power in this analysis.

Because only age, gender, and BMI were assessed at registration, we cannot exclude that other factors which were not investigated, such as education or socioeconomic status, may have influenced the results. The user’s socioeconomic status, in particular, might be of great interest for incentive participation and adherence as users with lower income are more likely to be appealed by the incentives [19] and are more adherent to interventions changing health behavior [12,45].

### Conclusions

This examination points out that financial incentives in the form of cashback or health credits have the potential to increase program adherence in a web-based PA intervention. Incentives are likely to act as an external motivator that enhances program adherence, which in turn can be the basis for a more intrinsically motivated, longer-lasting PA behavior in real life after the program has ended (behavior adherence). Therefore, health care providers may encourage program participation by providing differentiated incentives depending on gender, age, or BMI in web-based interventions promoting PA. Further investigations are needed to support these findings; in particular, it is necessary to further clarify the moderating role not only of gender, age, or BMI, but also of other potentially relevant factors such as socioeconomic status or education. The study results are based on naturalistic data with a high ecological validity; in the next step, it is necessary to confirm these findings in more controlled studies (eg, randomized controlled trials) with higher levels of internal validity.

## Appendix

Multimedia Appendix 1 Dashboard Fitness Coach mobile.

Multimedia Appendix 2 Knowledge Section Fitness Coach.

## Abbreviations

OR: odds ratio
PA: physical activity
