# Supplementary figures and images for: Effects of Incentives on Adherence to a Web-Based Intervention Promoting Physical Activity: Naturalistic Study
Source: J Med Internet Res. 2020 Jul 30;22(7):e18338. doi: 10.2196/18338 (PMC7426800; doi:10.2196/18338)

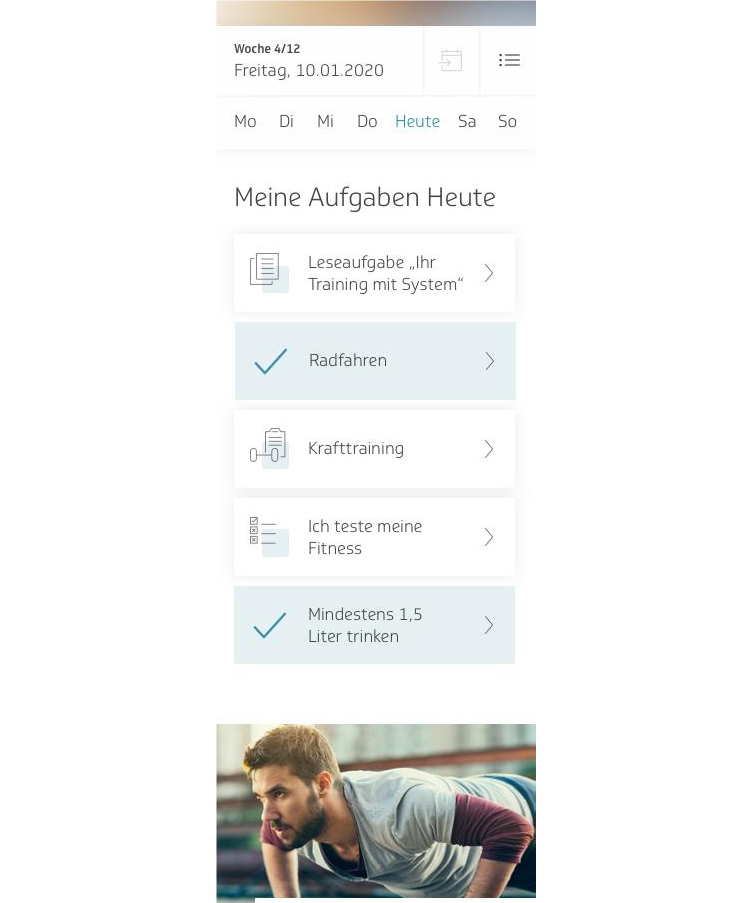

Supplement: Multimedia Appendix 1 [file jmir_v22i7e18338_app1.png]

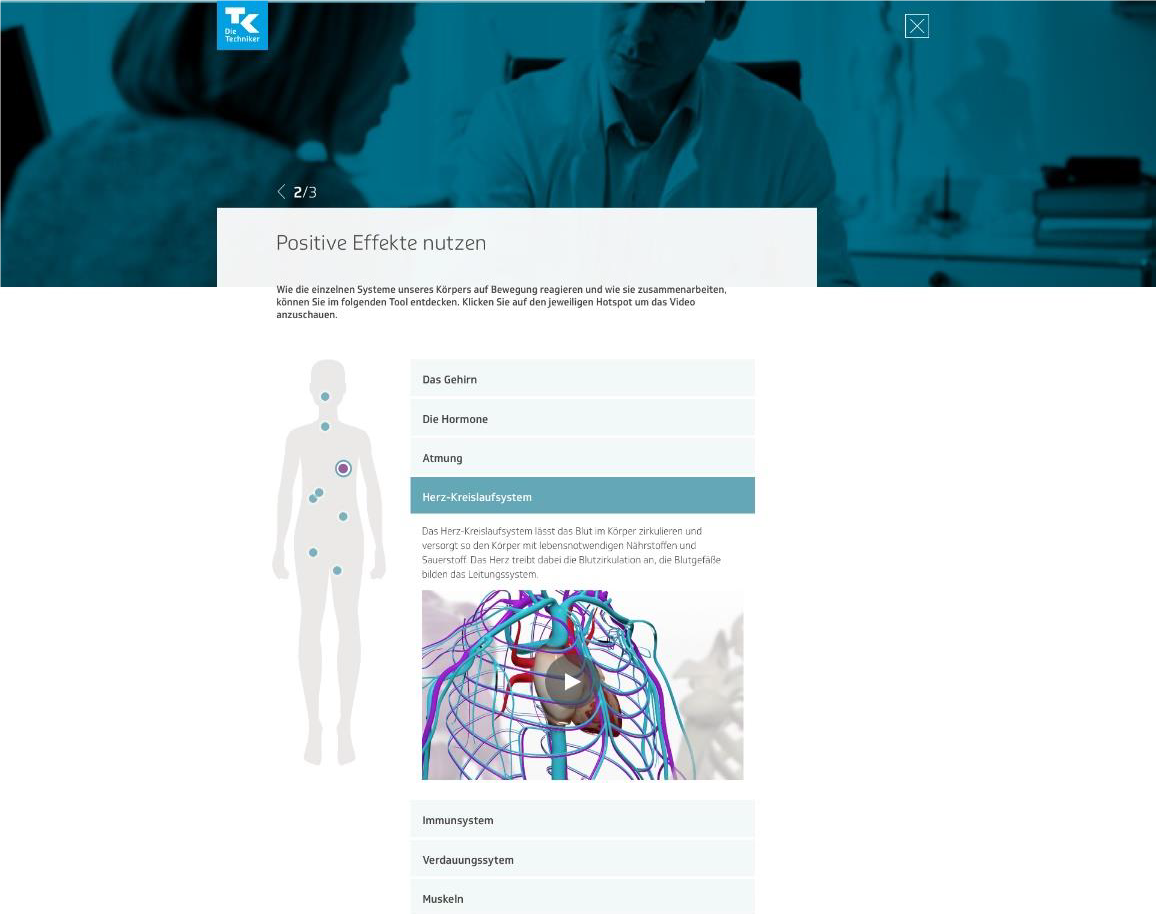

Supplement: Multimedia Appendix 2 [file jmir_v22i7e18338_app2.png]
